# Prospects and Challenges of Translational Corneal Bioprinting

**DOI:** 10.3390/bioengineering7030071

**Published:** 2020-07-06

**Authors:** Matthias Fuest, Gary Hin-Fai Yam, Jodhbir S. Mehta, Daniela F. Duarte Campos

**Affiliations:** 1 Department of Ophthalmology, RWTH Aachen University, 52074 Aachen, Germany; 2 Department of Ophthalmology, University of Pittsburgh, Pittsburgh, PA 15260, USA; yamg@pitt.edu; 3 Tissue Engineering and Stem Cell Group, Singapore Eye Research Institute, Singapore 169856, Singapore; jodmehta@gmail.com; 4 Singapore National Eye Centre, Singapore 169856, Singapore; 5 Institute of Applied Medical Engineering, RWTH Aachen University, 52074 Aachen, Germany; 6 DWI Leibniz Institute for Interactive Materials, 52074 Aachen, Germany

**Keywords:** bioprinting, corneal tissue engineering, hydrogel, cell-biomaterial interaction, 3D

## Abstract

Corneal transplantation remains the ultimate treatment option for advanced stromal and endothelial disorders. Corneal tissue engineering has gained increasing interest in recent years, as it can bypass many complications of conventional corneal transplantation. The human cornea is an ideal organ for tissue engineering, as it is avascular and immune-privileged. Mimicking the complex mechanical properties, the surface curvature, and stromal cytoarchitecure of the in vivo corneal tissue remains a great challenge for tissue engineering approaches. For this reason, automated biofabrication strategies, such as bioprinting, may offer additional spatial control during the manufacturing process to generate full-thickness cell-laden 3D corneal constructs. In this review, we discuss recent advances in bioprinting and biomaterials used for in vitro and ex vivo corneal tissue engineering, corneal cell-biomaterial interactions after bioprinting, and future directions of corneal bioprinting aiming at engineering a full-thickness human cornea in the lab.

## 1. Corneal Transplantation and Tissue Engineering

### 1.1. Bioarchitecture and Physiology of the Human Cornea

**Figure 1:**
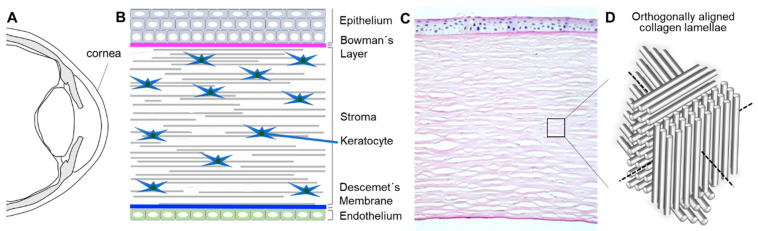
Bioarchitecture of the human cornea. Schematic illustration of (**A**) the cross-section of the human eye ball, (**B**) cornea, and (**C**) histological section of a human cornea showing the ultrastructure of the three main layers (epithelium, stroma, and endothelium) as well as the Bowman’s layer and the Descemet’s membrane (courtesy of Matthias Fuest, MD, RWTH Aachen University). (**D**) Illustration of orthogonally aligned collagen lamellae present in the human corneal stroma.

The human cornea, approximately 550 µm thick in the center and 650 µm in the periphery, is a transparent, dome-shaped tissue covering the front of the eye (Figure 1A) [1]. It serves three fundamental functions: (1) a mechanical and chemical barrier protecting the inner eye; (2) high transparency for light transmission; and (3) light refraction. Corneal clarity is due to the keratocytes that biosynthesize crystallins and organize regularly arranged collagen lamellae, relative avascularity as well as corneal dehydration that is regulated by corneal endothelial cells (Figure 1) [1].

**Figure 2:**
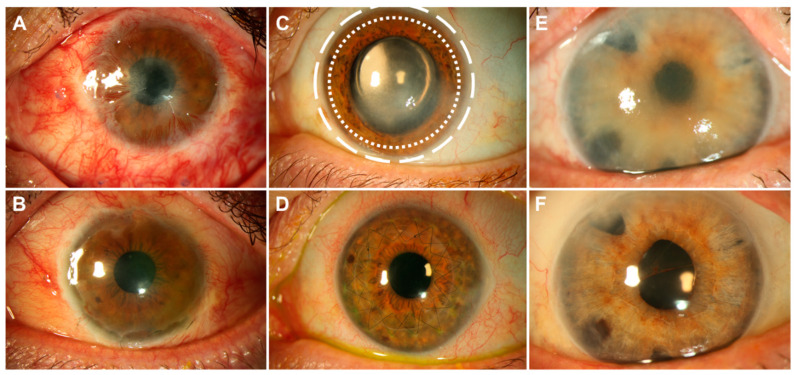
Eyes with diseases of the three different layers of the cornea before and after treatment. The crucial circular limbal area, host to most corneal stem cells, is located between the dotted white circles shown in (**C**). Patient (**A**) suffered a chemical burn to the surface of the eye that killed all epithelial stem cells, leading to a conjunctivalization of the cornea. The patient was treated with a limbal transplant from the healthy collateral eye ((**B**), 3 months post-surgery). Patient (**C**) had a stromal corneal scar following Herpes keratitis and underwent full thickness corneal transplantation ((**D**), 4 months post-surgery). Corneal endothelial cells of patient (**E**) died after a complicated cataract surgery, which led to swelling of the cornea and impaired vision. The eye underwent a lamellar endothelial corneal transplantation (Descemet membrane endothelial keratoplasty (DMEK), (**F**), 6 months post-surgery). All images are courtesy of Matthias Fuest, MD, RWTH Aachen University.

The corneal epithelium is a non-keratinized stratified squamous epithelium with 5–7 cell layers (Figure 1B). The superficial corneal epithelial cells (CEpCs) are flat and polygonal, connected with desmosomes and tight junctions as a permeability barrier, prohibiting the entering of foreign molecules and tears into the intercellular spaces. The basal layer consists of cuboidal or columnar CEpCs which are mitotically active to supply cells to the superficial layers. The symbiotic relationship of CEpCs with the overlying tear film provides a smooth regular surface with anti-microbial effects to maintain ocular health and clear vision [1]. Limbal stem cells (LSCs) that reside in the palisades of Vogt of the peripheral cornea (Figure 2C, white circles) continuously regenerate CEpCs. Damage to this region can lead to irreversible LSC deficiency (LSCD), resulting in impaired regeneration of CEpCs and keratopathy (Figure 2A,B) [2,3].

The stroma lies underneath the epithelium and accounts for about 90% of the corneal thickness. Its transparent and biomechanical characteristics result from the unique arrangement of collagen lamellae (predominantly I and V) and extracellular matrix (ECM) produced, organized, and maintained by corneal stromal keratocytes (CSKs). There are about 70 microfibrils within each collagen fibril of approximately 30 nm diameter. The fibrils are aligned and equally spaced to form a lamella of around 2 µm width, or 2000 nm. Around 250 collagen lamellae constitute the thickness of the central stroma of an adult human cornea. The lamellae are arranged orthogonally to each other to reduce light scatter (Figure 1D). Corneal stromal keratocytes are flattened dendritic cells, 31 µm wide and about 1 µm thick, predominantly disposed in the interface between adjacent lamellae. They produce and reorganize the ECM, secrete collagen and proteoglycans (keratan sulfate proteoglycan (KSPG); lumican, keratocan, and mimecan) which can influence the fibril spacing and optimize the optical characteristics of the cornea [1,4]. In a single interface, the cell bodies are well spaced across the cornea, but their thin, lengthy processes are so extensive (up to 100 µm) that they interdigitate with processes from neighboring cells, forming a delicate wide-mesh network (Figure 1). At intervals, CSKs project long, attenuated translamellar processes in anterior or posterior directions [5]. These can extend 30–50 µm from the cell body. Thus, an individual cell can bridge a corneal distance of about 100 µm, which is unusual for a non-neural cell but useful to maintain a transcorneal communicating network [6].

Corneal stromal keratocytes remain quiescent throughout adult life with minimal mitosis. However, infection or injury can wound the stroma which causes CSKs at the injured site to die, while the surviving CSKs at the wound periphery are “activated” to assume a repair cell type, the stromal fibroblasts (SFs). They are proliferative and motile cells with an attenuated expression of CSK genes and proteins (including keratocan, aldehyde dehydrogenase (ALDH), transketolase) but display a fibrosis-related gene profile (including fibronectin, tenascin, SPACR, and metalloproteinases) [4]. Some SFs further differentiate into myofibroblasts (MyoSFs, expressing, for example, alpha smooth muscle actin (SMA)), which produces abnormal ECM and induces tissue contraction, resulting in scar formation and corneal opacities, leading to vision loss (Figure 2C,D) [7,8]. Corneal stromal stem cells (CSSCs) have been identified within the limbal stroma [9].

The monolayered corneal endothelium regulates the stromal hydration maintaining corneal transparency [10]. A continuous ATPase activity of the corneal endothelial cells (CECs), facilitates the fluid-coupled active transport of ions from the corneal stroma to the aqueous humor. This pumping activity prevents the overhydration (edema) of the stroma, facilitating corneal transparency [10]. Although progenitors are suspected to reside in the posterior limbal area [11,12], adult human corneal endothelial cells (hCECs) do not proliferative in vivo. Aging, trauma or iatrogenic factors cause cell loss, leading to corneal edema and deterioration of vision (Figure 2E,F). Corneal endothelial disorders currently represent the most common reason for corneal transplantation in industrialized countries [13].

### 1.2. State-of-the-Art Corneal Transplantation

Corneal transplantation remains the preferred treatment option for advanced stages of stromal and endothelial diseases. Even though tremendous advancement in surgical technique has been made in the last twenty years, many factors remain, that limit long-term success, such as global scarcity of donor material, high costs, limited graft survival, allogeneic graft rejection, the need for immunosuppressants, prolonged post-surgery management, and the need for highly trained surgeons to perform the procedures [13,14].

Despite the increasing total number of donors in the last years, the growth of the global population and the demographic trends will exacerbate the worldwide issue of donor material shortage. Even in regions with well-developed eye banking systems, e.g., USA and Western Europe, many potential donor corneas have to be eliminated because of the positive testing for transmissible viruses (like hepatitis B and C) [15]. Additional aspects, such as medication history or religious beliefs, further reduce the donor pool. All these factors promote the search for suitable engineered tissue substitutes.

### 1.3. State-of-the-Art Corneal Tissue Engineering Strategies

Corneal tissue engineering can bypass many complications of conventional corneal transplantation and has gained increasing attention recently. The human cornea is avascular and immune privileged. It is therefore an ideal organ for tissue engineering, as rejection and inflammatory responses occur less likely than in other locations [16]. However, the complex nature of corneal tissue to maintain clarity and biomechanical stability as well as the bio-integration of the engineered substitutes remains the main challenge. Prior strategies include the construction of the ECM, the cells or a combination of both for the epithelial, stromal or endothelial layers [17]. Few groups have targeted more than one layer.

**Epithelium:** Corneal epithelial cells proliferate continuously from the limbus onto the cornea and also onto centrally implanted tissue-engineered corneal substitutes [2,3]. In case of insufficient migration or adherence, the surface of the construct can be optimized by coating with collagen, laminin, fibronectin, or fibrin [18]. In recent years in corneal epithelial tissue engineering, much effort was given to searching for biocompatible, mechanically stable, and optically transparent substrates that allow efficient cell adhesion, migration, and proliferation for the ex vivo expansion of LSCs to treat LSCD [18,19,20]. To date, human amniotic membrane (hAM) has been the most commonly used biological matrix, due to the fac of its inherent growth factor content, the ability to facilitate epithelialization, its low immunogenicity, antifibrotic, antiangiogenic, antimicrobial, and antiviral properties [19,21]. However, hAM has several limitations. The theoretic possibility of disease transmission, variation of growth factor content depending on the storage method, [22] and donor characteristics, [23] the processing following caesarean section is demanding, particularly to avoid bacterial contamination, [24] and, ultimately, the exact mechanism of action remains unclear [21]. For these reasons, and given the variable quality of this material and its reduced transparency, [18] new studies focused on the investigation of biological and synthetic alternatives. For example, collagen constructs promoted CEpC growth in vitro and in animal models [18]. Nevertheless, the high water content limits the stability of collagen hydrogels which can be increased by either mechanical compression or chemical crosslinking [20]. Fibrin sealant was used as a substrate for the expansion of LSCs in the treatment of more than 113 patients with LSCD [25,26]. Optically robust, highly permeable, and elastic, corneal constructs have been produced from tropoelastin–silk films, supporting CEpCs and CECs in vitro [27]. Human anterior lens capsules supported LSCs; however, the availability and fragility limited its clinical application [28]. Human hair keratin films showed high light transmission, yet suture loosening resulted in poor anchorage to the ocular surface [29]. Human LSCs could successfully be cultivated on glutaraldehyde cross-linked chitosan–gelatin combinations. Scaffolds with higher gelatin content degraded more rapidly, whereas those with a higher chitosan content were more stable but also more brittle [30]. Ocular surface reconstruction in vivo with these constructs is yet to be tested.

Synthetic polymers offer standardized mass-production and lack of biological material, thus obviating concerns of disease transmission. Results for FDA-approved polymers, such as poly(lactide-co-glycolide (PLGA), hydroxymethylacrylate (HEMA), and polymethacrylate carriers, are limited to in vitro studies thus far [31,32]. In rabbit models, implants made from polyethylene glycol (PEG) and polyacrylic acid (PAA) led to corneal inflammation, haze, and ulceration [33,34]. Further investigations are needed to evaluate their potential.

**Stroma:** In corneal stromal tissue engineering, different components have been tested similarly as for epithelial tissue engineering. Collagenous materials with crosslinking or mechanical compression have shown improved construct stability but limitations in cell viability and matrix remodeling [20,35]. Collagen vitrigels contain a high proportion of water which renders them intrinsically weak, unless blended with other polymers, to create collagen composites, or chemical crosslinkers. Modification can limit the direct seeding of cells within the scaffold. However, they do promote dendritic branching, cell elongation, and CSK expression of keratocan and ALDH in vitro [36]. Other substances, that have been investigated as alternative stromal biomaterials, include PLGA, gelatin, and chondroitin sulfate [37,38,39]. Cell-free corneal implants comprising recombinant human collagen and phosphorylcholine were recently grafted by anterior lamellar keratoplasty into corneas of unilaterally blind patients diagnosed at high-risk for transplant rejection. Grafting promoted nerve regeneration as observed by improved touch sensitivity in all and vision in three out of six patients [39].

Many corneal stromal tissue-engineered constructs have difficulties reaching the demands in tensile strength, natural surface curvature, and stromal architecture of the human cornea which can result in reduced optical transparency. Nevertheless, promising approaches to addressing these issues have recently been published. The use of curved templates helped to generate more realistic corneal curvatures, and facilitated CSK and collagen alignment, leading to improved elastic modulus [40]. Using peptide amphiphile-coated surfaces with different anisotropies and cultured human CSKs, Gouveia et al. created corneal stromal self-lifting analogous tissue equivalents (SLATEs) [41]. These SLATEs comprising aligned collagen fibrils, hence imitating the microarchitecture of the human cornea, were transparent and thicker, denser, as well as more resistant to proteolytic degradation compared to SLATEs formed with randomly oriented constituents. The constructs also integrated well in a rabbit corneal model. In another study, Miotto et al. [42] were able to induce self-curving of collagen-based hydrogels via contraction-inhibiting peptide amphiphiles in certain regions of the gels. However, the contraction was facilitated by alpha SMA expressing corneal stromal cells (i.e., Myo-SFs), which can cause corneal scarring and reduced transparency. Ghezzi et al. [43] used patterned, porous, thin, and optically clear silk protein films stacked in an orthogonally, multi-layered architecture resembling the human cornea, seeded with human CSSCs, to generate 3D functional corneal stroma tissue equivalents. The cells secreted characteristic ECM and remodeled the constructs. Another promising approach are decellularized corneas (DC) from animal and human origins, as they retain the prevailing three-dimensional ECM structure, biomechanics, biocompatibility, and transparency [44]. The complete removal of all cell remnants is essential to reduce immunogenicity, and the preservation of the ECM ultrastructure enables an efficient recellularization and high biocompatibility [45]. To date, different protocols to decellularize entire corneas or thin stromal lenticules are promoted and there is no agreement on the best approach [45]. The key difference in the protocols is the preservation of the crucial membranes in the tissue, i.e., Bowmans and Descemets with respect to decellularization efficiency [45,46,47]. Drawbacks that remain are the loss of ECM components, alterations of the stromal structure, and biomechanical stability as well as inability to allow a complete recellularization by the host’s CSKs post-implantation [45,46,47,48].

**Endothelium:** Human CECs in vivo are postmitotic and do not proliferate. However, in vitro, hCECs have been expanded and reinjected into the eyes of patients with endothelial disease [49]. Attachment was facilitated by face-down positioning of the patients. In analogy, hCECs can also be seeded on stromal constructs, biological matrices, e.g., gelatin, collagen I gels (vitrigel), gelma, [50] animal and human DC [51,52,53,54,55], or synthetic lamellae, e.g., PLGA, poly-L-lactic acid (PLLA), and chitosan [56,57]. These tissue engineered carriers were transplanted onto Descemet membrane-stripped recipient corneal stromal beds in different animal models [51,54]. Synthetic polymers provide high purity with known chemical composition, physical properties, structure, and degradation times. Nevertheless, some components can induce inflammatory reactions [57]. Biological carriers, particularly DC lamellae, advantageously represent the natural substrate for hCECs [58]. However, grafts can be rejected, especially in cases of insufficient decellularization and they may transfer infections. In addition to this, the use of carriers from human material does not reduce the dependency on donor tissue, even though multiple grafts could be engineered from one donor cornea. 

### 1.4. Drawbacks of State-of-the-Art Approaches and Future Directions

Nowadays, human LSCs and CECs can effectively be propagated ex vivo and re-implanted in patients, [26,49] as well as on tissue-engineered constructs [26,27]. Hence, for the creation of an entire artificial cornea much effort has to be given to the reconstruction of the human stroma, which warrants the most crucial corneal functions. As described in the previous section, most corneal tissue engineered constructs have difficulties to satisfy all the demands posed on acceptable substitutes of the human corneal stroma:

(a) **Biological function**: CSKs should show typical dendritic shapes, forming networks, and not differentiate into scar-inducing SF and Myo-SF phenotypes [59];
(b) **Transparency**: the human corneal light-transmittance rises rapidly from 300 nm, reaching 80% at 380 nm and more than 90% between 500 and 1300 nm [60];
(c) **Mechanical properties**: The biomechanical characteristics of the human cornea and, hence, the natural habitat of CSKs are complex. In short, different grades of stiffness can be found in the human cornea, depending on age, strain, and position. Ex vivo destructive testing has successfully confirmed the following biomechanical principles:
  (i) The cornea exhibits a non-linear stress versus strain response with progressive stiffening at high strains [61].
  (ii) The cornea shows regional in-plane variation in strain and deformation, meaning that the paracentral and peripheral cornea is stiffer than the central cornea due to the differing orientation and number of collagen fibrils [62].
  (iii) Corneal elastic strength is a function of depth with decreasing strength from the anterior to the posterior stroma [63]. Young’s modulus of elasticity for the anterior human cornea (first 50 µm including Bowman’s lamella) was measured by indentation at 245 ± 209 kPa (range: 82–530 kPa), and for the posterior stroma at 100 ± 61 kPa (28–162 kPa) [64].
  (iv) Corneal mechanical properties are dependent on age, with corneal stiffness increasing with the age of the patient [65,66]. At physiological intraocular pressure of 15 mmHg, corneal stiffness was found between 200 (50–65 years) and 700 kPa (80–95 years).
(d) **Curvature**: The cornea has the highest refractive power of the human eye (approximately 43 diopters). The average radius of the anterior corneal surface measured by Scheimpflug imaging was 7.7 ± 0.2 mm, and the average radius of the posterior corneal surface was 6.5 ± 0.2 mm [67].

Considering these demands, automated biofabrication strategies, such as bioprinting, may offer additional spatial control during the manufacturing process to generate cell-laden 3D constructs comprising three layers. In the Section 2, we discuss recent advances in bioprinting and biomaterials used for in vitro and ex vivo corneal tissue engineering.

## 2. Bioprinting Methods for Tissue Engineering

**Figure 3:**
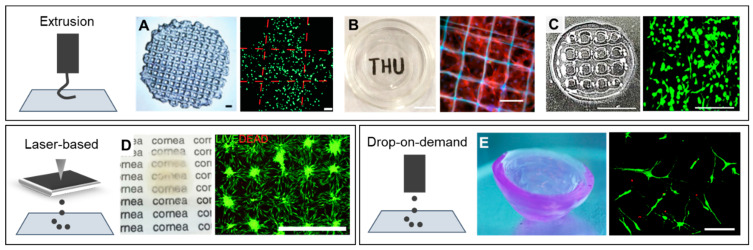
Cellular morphology in bioprinted corneal stromal tissue-like structures strongly depends on the material used as bioink. (**A**) Extrusion bioprinting of human corneal stromal keratocytes (CSKs) in 15% GelMa. Fluorescent staining shows round cells after bioprinting. Scale bars represent 1 mm (left) and 100 µm (right) [88]. (**B**) Extrusion bioprinting of rat limbal stromal stem cells in PEG-PCL reinforced 15% GelMa. Fluorescent staining shows cellular filopodial extensions up to 50 µm. Scale bars represent 10 mm (left) and 100 µm (right) [87]. (**C**) Extrusion bioprinting of human turbinate-derived mesenchymal stem cells cultured in CSK differentiation media and embedded in a cornea-derived decellularized ECM bioink. Cells show filopodial extension up to 50 µm. Scale bars represent 5 mm (left) and 200 µm (right) [89]. (**D**) Laser-based bioprinting of limbal epithelial cells and adipose-derived stem cells in a Matrigel-collagen type-I-based bioink. After bioprinting, cells can spread in the printed substrate. Scale bar represents 1 mm [84]. (**E**) Drop-on-demand bioprinting of human CSKs in 0.5% agarose–0.2% collagen type-I hybrid bioink. Cells are able to extend their filopodia up to 100 µm. Scale bar represents 100 µm [86].

Bioprinting is a key biofabrication strategy used to create artificial ex vivo tissues and organs by sequential deposition of cell-laden hydrogel layers [68,69]. Current bioprinting strategies can be categorized into extrusion-based, droplet-based, and laser-based approaches (Figure 3) [68]. Each printing method is compatible with one or more types of bioinks that crosslink in distinct ways. Therefore, large efforts have been made toward optimizing bioprinting methods for one single bioink. For example, extrusion bioprinting is by far the most frequently used bioprinting method [70,71,72]. Briefly, it consists of extruding thin strands of pre-polymerized bioinks through a needle into a printing platform by applying pressurized air to the printer head. This procedure can be done using more than one printer head, and it is repeated layer by layer until a 3D construct is generated. Extrusion bioprinting is the preferred method among biomedical scientists, given that it is compatible with a wide range of injectable hydrogels developed for regenerative medicine applications [73,74,75]. So far, interesting examples have demonstrated the promise of bioprinting to create ex vivo tissues and disease models. For instance, researchers have used microextrusion bioprinting to generate expansion lattices for neural research, [76] laser-based bioprinting to construct 3D co-culture models of interacting cancer and endothelial cells (ECs) [77] as well as drop-on-demand bioprinting to recreate the native environment of articular cartilage [78].

Besides these three classic bioprinting methodologies, recent studies introduced four new variations that have shown encouraging results. From these studies, two focused on the use of photopolymerizable gels [79,80], and the other two were compatible with natural, thermosensitive bioinks [81,82]. The first two studies employed hydrogels with similar crosslinking mechanisms as a basis for their bioink (gelatin methacrylate, gelMA; and poly(ethylene glycol) diacrylate, PEGDA). Both materials can initiate crosslinking when combined with photoabsorber additives, such as lithium phenyl (2,4,6-trimethylbenzoyl)phosphinate (LAP), in the presence of visible light. In the work of Bernal et al. [79], a fully printed osteogenic model was generated by volumetric bioprinting. Using this method, a gelMA-LAP container was set into rotation and synchronously irradiated with a sequence of 2D light patterns. The polymer solidifies only in selective areas where the accumulation of multiple angular exposures results in an absorbed dose overcoming the gelation threshold. The 3D-printed models showed increased expression of alkaline phosphatase and mineral deposition after 14 days in culture. In the work of Grigoryan et al. [80] intravascular and multivascular design freedoms were generated with photopolymerizable hydrogels by using food dye additives as biocompatible yet potent photoabsorbers for projection stereolithography. A printed distal lung subunit model was generated, which was composed of a concave and convex airway ensheathed in vasculature and responded to tidal ventilation and oxygenation upon perfusion with blood. Both studies showed that it was possible to generate very intricate 3D structures in the micrometer scale. Therefore, these bioprinting methods may open new avenues for fabricating ex vivo tissues, such as bone, and respiratory epithelium, and eventually be translated for applications in ophthalmology research. The other two recent bioprinting methods, which are compatible with natural, thermosensitive hydrogels, were proposed by the Lewis group [81] and the Feinberg group [82]. The study by Skylar-Scott et al. [81] introduced, for the first time, a sacrificial writing into functional tissue (SWIFT) bioprinting to generate diagonal vascular branches into a cardiomyocyte/fibroblast-laden bioink mimicking the left anterior descending artery. In SWIFT, millions of cell aggregates are transferred to an ECM-mimicking reservoir that remains in a liquid-like state during the bioprinting process. A flat-tip needle coupled to a microextruder containing a sacrificial gel, such as gelatin, is positioned into the cell aggregate reservoir and prints a vascular pattern. The printed sacrificial gel is then liquefied at 37 °C and removed after the printing process, allowing for medium flow through the 3D construct. In this study, a perfusable cardiac tissue was generated with vascular-like channels with a diameter of about 1 mm. After bioprinting and eight days of culture, spontaneous cardiomyocyte beating was observed, demonstrating the functionality of these artificial tissues despite the printing process. The opposite concept of sacrificial bioprinting was employed in the study by Lee et al. [82]. Here, freeform reversible embedding of suspended hydrogels (FRESH) bioprinting was used to bioprint an organ-scale neonatal heart with multiscale vasculature. The FRESH method utilized a sacrificial gelatin-based gel, which supported the shape of fragile cardiomyocyte-laden collagen bioinks during the 3D assembly process. Encouragingly, after fourteen days of culture, the printed neonatal heart was able to generate electrophysiological spontaneous contractions. These findings altogether may be used to design and construct human corneal substitutes, which also predominantly contain collagens and other elastic fibers in vivo. To summarize, bioprinting is a very promising methodology to reconstruct complex tissues, such as cornea, because of the possibility to automatically, sequentially construct different layers with customized architecture and biological and biomechanical properties that can further be loaded with characteristic cell types. Other approaches usually require numerous manual steps prone to human error.

## 3. Corneal Bioprinting: Focus on Stroma

As introduced earlier in Section 1.1, there are five distinct layers that compose the human cornea. The thickest layer is the stroma (~500 µm), and the remaining four layers (epithelium, Bowman’s layer, Descemet’s membrane, and endothelium) are delicate structures <50 µm in thickness. The stroma comprises approximately 90% of the corneal tissue and is the most crucial structure in corneal tissue engineering, due to the fact of its high demands in terms of biomechanical stability (cross-linked collagen lamellae), and transparency (arrangement of collagen fibrils, proteoglycans, and the presence of crystallins) [1]. Also, the central and peripheral parts of the cornea present a distinct stiffness, which influences the orientation of collagen fibrils as well as orientation and differentiation of corneal cells [83]. In order to maximize the potential of bioprinting in a corneal tissue engineering context, it is crucial to recapitulate the macro and microstructure of the stromal layer compared to the other parts with rather plain geometries (endothelium and epithelium). As the stroma has very characteristic stiffness, curvature, and microstructure depending on biological factors as mentioned in previous sections, in this section we focus on recent advances in corneal stromal bioprinting.

Several bioprinting approaches for corneal tissue engineering have been investigated [84,85,86]. Sorkio et al. [86] introduced a laser-based bioprinting technique to create corneal mimicking structures (Figure 3D). Briefly, a donor slide was coated with a thin laser-absorbing laminin-collagen type-IV layer with a thicker layer of a cell-laden collagen type-I-based bioink and placed upside-down in the printing setup. Laser pulses were focused through this donor slide creating expanding vapor bubbles that were collected at the collector slide with arbitrary geometric designs. Seven days after printing, the cultured substrates showed increased cell proliferation using human LSCs and human adipose-derived stem cells in comparison to day one cultures. Despite the successful feasibility to print such geometries, CSKs are a very sensitive cell type, and the substrate could not demonstrate the integration of CSKs into gels, hence questioning its suitability for its application as a corneal implant. Moreover, the mechanical properties, microstructure, and geometrical curvature of these printed specimens were not addressed, and qualitative visual examination showed slightly opaque constructs. In the study of Isaacson et al. [85] a digital model from a patient’s cornea was used as a template to print a 3D mold made of acrylo-nitrile butadiene styrene (ABS). Human CSKs were loaded in an alginate-collagen type I co-gel and extruded onto the previously generated 3D model, and simultaneously supported by FRESH during the printing process. The printed constructs showed 3D geometries similar to that of native corneas. Despite this significant advance, CSKs contained in the 3D-printed constructs remained rounded during the post-printing culture and were not able to elongate into a dendritic shape similar to CSKs shape in vivo. The printed constructs were qualitatively transparent. Unfortunately, the mechanical stability and microstructure of these specimens was not analyzed in this study.

Isolation and propagation of CSKs is challenging, as this delicate cell type easily transforms to scar inducing SFs. This challenging aspect was later improved in a recent study, that employed drop-on-demand bioprinting, [86] which is a more cell-friendly printing method relative to extrusion and laser-based bioprinting. In this study, CSKs were encapsulated in agarose-collagen type I bioinks and printed through a microvalve that was coupled to a pressurized air supply. 3D constructs with dome-shape geometries were printed drop-by-drop and layer-by-layer without a supporting structure or fluid (Figure 3E). Corneal stromal keratocytes contained in these printed constructs showed similar viability as non-bioprinted controls. Importantly, printed CSKs stained positive for keratocan and lumican, which are characteristic corneal stromal markers, and showed up to 50 µm long filopodia in printed 3D constructs. However, the mechanical stability of these constructs was only 15–20 kPa, and the light transmittance for different wavelengths was not assessed.

Regeneration of corneal stroma has always been an obstacle in tissue engineered approaches, due to its sophisticated microstructure, consisting of orthogonally aligned collagen nanofibrous lamellae [87]. The transparency and mechanical properties of the stroma are attributed to its unique tight packing and uniform diameter of collagen fibers. A recent study fabricated orthogonally aligned PCL-PEG sub-micro fibers, which were perfused by rat LSC-loaded 15% GelMA hydrogel, to form 3D fiber-reinforced constructs (Figure 3B) [87]. The combination of the PCL-PEG fibers with the 15% GelMA gel improved the strength of the constructs to address issues with the suture-ability. Corneal stromal keratocytes cultured in scaffolds printed with 100 µm fiber diameters showed high expression of CSK markers. These constructs were qualitatively transparent and showed average compressive modulus of 120 kPa. After intrastromal keratoplasty in rat corneas, these constructs showed high light transmittance and degradation of the scaffolds three months post-surgery. However, two eyes had severe inflammation after implantation, and four out of thirty eyes showed neovascularization of the corneas, detailed histopathological studies (i.e., immunohistochemistry) were not performed. The curvature of these specimens was also not addressed in this study. Other research groups have presented similar extrusion printing strategies for corneal stromal tissue engineering using either GelMa or decellularized corneal ECM (dECM) gels (Figure 3A,C) [88,89]. Corneal stromal keratocytes differentiated from human turbinate-derived mesenchymal stem cells, that were embedded in corneal dECM gels with higher concentrations (2 wt%), showed improved mechanical properties after culture, in relation to gels with lower concentrations (0.5 wt%). Cells contained in printed specimens showed filopodial elongations up to 50 µm [89]. In contrast, human CSKs cultured in 15% GelMa gels showed round geometries after printing and much lower mechanical stability (10–20 kPa) compared to corneal dECM bioinks (70–100 kPa) [88]. Corneal dECM bioinks seem to be advantageous for corneal stromal tissue engineering in relation to GelMa bioinks, given that lower concentrations of this bioink allow for both, improved mechanical properties in the range of 100 kPa, and cellular morphological features similar to CSKs in vivo. These results contribute to our understanding of human cell-material interactions in corneal bioprinting. Aspects like composition and concentration of bioinks should be considered for the success in printability, but also for their high impact on cell behavior e.g., cell elongation, phenotype maintenance, and remodeling in the 3D constructs.

Based on the studies presented thus far, it is challenging to drive conclusions on what is potentially the best manufacturing method to generate 3D corneal substitutes. Extrusion bioprinting allowed for the printing of constructs with improved mechanical properties (up to 120 kPa), whereas with drop-on-demand bioprinting constructs achieved elastic modulus up to 20 kPa. Studies that focused on laser-based bioprinting did not analyze the mechanical properties of the constructs. As reported in the literature, corneal stroma has an elastic modulus of about 150–700 kPa. Therefore, if the mechanical properties were to be alone the most important aspect for corneal tissue engineering, extrusion bioprinting would be given preference. However, other parameters should also be considered, including curvature and microstructure. The best curvature results were shown by drop-on-demand bioprinting. Bioprinted constructs using this technology neither require the use of molds during printing nor the use of supporting hydrogels like FRESH. It is an advantage to use bioprinting to recreate the native curvature of the human cornea, compared to casting hydrogels in a mold, because it is an automated, freeform process that does not rely on manual replication prone to human error. With regards to the microstructure, i.e., the microarchitecture, of fibers and cells aligned within the constructs, so far none of the presented studies addressed this aspect in combination with bioprinting. In a recent study, researchers used poly-tetrafluoroethylene nanogrooves to direct the orientation of corneal stromal cells in manually casted specimens [40]. Future corneal bioprinting research may combine principles from both technologies i.e., bioprinting and smart fiber alignment technologies in order to automatedly generate constructs with microarchitectures mimicking that of native corneas. In short, future approaches may need to combine the best attributes from distinct technologies to create hybrid manufacturing methods that enable the production of man-made, lab-grown corneal tissues.

A summary of all studies found in the literature until 2020 on corneal stromal bioprinting, as well as studies on endothelial and epithelial corneal bioprinting are listed in Table 1, including the type of bioprinting method, material as bioink, mechanical properties, and cell features being examined in each study.

## 4. Future Directions of Corneal Bioprinting: Full-Thickness Human Corneas

The main goal in human corneal tissue engineering is to create a full-thickness artificial cornea with the reconstruction of the complex stroma being the most demanding part. To summarize the results presented thus far, none of the bioprinting approaches has been able to satisfy all demands. Some studies show high survival rates and elongation of human CSKs but suboptimal mechanical properties [86]. Others were able to produce constructs with a high light transmission, yet low biomechanical stability and lack of cell–cell interactions [88]. Future developments in 3D bioprinting have to optimize the technology and components to match the complexity of the human corneal stroma.

As introduced in the beginning of this article, human corneas are made of five distinct layers. Given that corneal stromal bioprinting has shown encouraging results up to now, one can consider expanding this knowledge to manufacturing in vitro the remaining layers of the cornea, despite their distinct characteristics relative to the stroma. Two examples of studies that experimented with epithelial and endothelial corneal bioprinting are listed in Table 1 [90,91]. In the study by Zhang et al. [90], human CEpCs were embedded in 15% GelMa hydrogels and printed by extrusion. In the other study by Kim et al. [91], human CECs were embedded in a gelatin–RGD bioink and printed in an amniotic membrane support. In both studies, cells remained round inside the matrices after printing and did not form mono/multilayers, questioning the choice of materials or the need for printing these very thin layers with an automated dispensing mechanism. In the human cornea, CEpCs form a non-keratinized stratified squamous epithelium. These cells have a hexagonal shape and form a tight monolayer. As human CEpCs generally proliferate and migrate from an intact limbus onto the cornea, one could hypothesize that these cells could migrate onto a bioprinted corneal construct without further need to fabricate in vitro an additional epithelial layer. Contrastingly, engineering the corneal endothelium in vitro might be beneficent, given that human CECs in vivo are postmitotic and do not proliferate. For this reason, CECs could be added in solution to form a monolayer next to the corneal stroma 3D construct after bioprinting.

## Figures and Tables

**Table 1:** Summary of original studies performed in 2018–2020 on corneal bioprinting.

Corneal Layer	Bioprinting Method	Materials for Bioink	Cell Type	Cell Elongation	Mechanical Properties	Transparency Evaluation	Reference
**Native cornea**							[60]
Stroma			CSKs/LSSCs	Dendritic up to 100 µm	100–250 kPa	80% at 380 nm and	
Epithelium			CEpCs/LECs	Round/polygonal tight monolayer	Fragile	>90% at 500–1300 nm	
Endothelium			CECs	Hexagonal tight monolayer	Fragile		
**Stroma**	Laser	Matrigel-COL I bioink // LN-COL IV support sheet	Human LECs+ ADSCs	Filopodial elongation up to 50 µm	n.a.	Qualitative: slightly opaque	[84]
	Extrusion	1.3% ALG-2.7% COL I bioink // FRESH support	Human CSKs	Round cells	n.a.	Qualitative: see through gel	[85]
	Drop-on-demand	0.2% COL I-0.5% AG bioink // no support	Human CSKs	Filopodial elongation up to 100 µm	15–20 kPa	Qualitative: see through gel	[86]
	Extrusion	15% GelMa bioink // reinforced with PEG-PCL fibers	Rat LSSCs	Filopodial elongation up to 50 µm	60–120 kPa	Qualitative: see through gel	[87]
	Extrusion	15% GelMa bioink // no support	Human CSKs	Round cells	10–20 kPa	Quantitative: 80% at 700 nm 5% at UVB	[88]
	Extrusion	Cornea-derived dECM bioink // no support	Human TDMSCs with keratocyte induction	Filopodial elongation up to 50 µm	70–100 kPa	Quantitative: 80% at 700 nm 70% at UVB	[89]
**Epithelium**	Extrusion	15% GelMa bioink // 15% GelMa dome-shaped mold	Human CEpCs line	Round cells	50 kPa	Qualitative: see through gel	[90]
**Endothelium**	Extrusion	Gelatin-RGD bioink // amniotic membrane dECM support	Human CECs	Round cells	n.a.	n.a.	[91]

Abbreviations: collagen (COL), laminin (LN), alginate (ALG), free-form reversible embedding of suspended hydrogels (FRESH), agarose (AG), methacrylated gelatin (GelMa), decellularized extracellular matrix (dECM), limbal epithelial cells (LECs), adipose-derived stem cells (ADSCs), corneal stromal keratocytes (CSKs), limbal stromal stem cells (LSSCs), turbinate-derived mesenchymal stem cells (TDMSCs), corneal epithelial cells (CEpCs), corneal endothelial cells (CECs).
